# Cysteine and Methionine Biosynthetic Enzymes Have Distinct Effects on Seed Nutritional Quality and on Molecular Phenotypes Associated With Accumulation of a Methionine-Rich Seed Storage Protein in Rice

**DOI:** 10.3389/fpls.2020.01118

**Published:** 2020-07-22

**Authors:** Sarah J. Whitcomb, Apidet Rakpenthai, Franziska Brückner, Axel Fischer, Saroj Parmar, Alexander Erban, Joachim Kopka, Malcolm J. Hawkesford, Rainer Hoefgen

**Affiliations:** ^1^Laboratory of Amino Acid and Sulfur Metabolism, Department of Molecular Physiology, Max Planck Institute of Molecular Plant Physiology, Potsdam, Germany; ^2^Bioinformatics Infrastructure Group, Max Planck Institute of Molecular Plant Physiology, Potsdam, Germany; ^3^Plant Sciences Department, Rothamsted Research, Harpenden, United Kingdom; ^4^Applied Metabolome Analysis Infrastructure Group, Max Planck Institute of Molecular Plant Physiology, Potsdam, Germany

**Keywords:** methionine, cysteine, nutritional quality, seed storage protein, serine acetyltransferase, cystathionine gamma-synthase, endoplasmic reticulum, *Oryza sativa*

## Abstract

Staple crops in human and livestock diets suffer from deficiencies in certain “essential” amino acids including methionine. With the goal of increasing methionine in rice seed, we generated a pair of “Push × Pull” double transgenic lines, each containing a methionine-dense seed storage protein (2S albumin from sunflower, HaSSA) and an exogenous enzyme for either methionine (feedback desensitized cystathionine gamma synthase from Arabidopsis, AtD-CGS) or cysteine (serine acetyltransferase from *E. coli*, EcSAT) biosynthesis. In both double transgenic lines, the total seed methionine content was approximately 50% higher than in their untransformed parental line, *Oryza sativa* ssp. *japonica* cv. Taipei 309. HaSSA-containing rice seeds were reported to display an altered seed protein profile, speculatively due to insufficient sulfur amino acid content. However, here we present data suggesting that this may result from an overloaded protein folding machinery in the endoplasmic reticulum rather than primarily from redistribution of limited methionine from endogenous seed proteins to HaSSA. We hypothesize that HaSSA-associated endoplasmic reticulum stress results in redox perturbations that negatively impact sulfate reduction to cysteine, and we speculate that this is mitigated by EcSAT-associated increased sulfur import into the seed, which facilitates additional synthesis of cysteine and glutathione. The data presented here reveal challenges associated with increasing the methionine content in rice seed, including what may be relatively low protein folding capacity in the endoplasmic reticulum and an insufficient pool of sulfate available for additional cysteine and methionine synthesis. We propose that future approaches to further improve the methionine content in rice should focus on increasing seed sulfur loading and avoiding the accumulation of unfolded proteins in the endoplasmic reticulum.

*Oryza sativa* ssp. *japonica*: urn:lsid:ipni.org:names:60471378-2.

## Introduction

Unlike plants, all animals lack the enzymatic machinery to synthesize *de novo* some of the 20 proteinaceous amino acids. These so called “essential amino acids” must be consumed in their diet. Further, for optimal growth, these essential amino acids must be consumed in the right balance for the animal’s metabolic needs. Amino acids that are in excess of amount defined by the first limiting amino acid will be catabolized, and the effective protein content of the feed will be reduced.

Methionine (Met) is one such essential amino acid, but the amount present in plant-based animal feed blends is typically insufficient optimal livestock growth and health. Cysteine (Cys) is not strictly considered an essential amino acid in the diets of animals because it can be synthesized from methionine, but in dietary situations where methionine is limited, cysteine becomes conditionally essential. Increasing the methionine and cysteine content of commodity cereals and grain legumes would benefit farmers by elevating the value of their crop and would be of benefit to livestock rearing by reducing the need for synthetic amino acid supplementation of animal feed.

Crops differ dramatically in how they store methionine. For example, in potato tubers 90% of the methionine is soluble (Dancs et al., 2008), while in alfalfa leaves (Amira et al., 2005) and cereal grains (Amir et al., 2018) almost all methionine is incorporated into protein. In tissues such as seeds that store Met predominantly in protein, a relatively direct approach to elevate the Met content is to increase the protein sink strength fraction by introducing genes for methionine-rich seed storage proteins (SSP). This approach assumes that the sink strength for methionine in endogenous seed proteins is relatively low, and this limits methionine accumulation in the seed. As seeds contain only low levels of free amino acids (Amir et al., 2018), this approach also assumes that the synthesis and/or metabolism of methionine is sensitive to signaled demand from SSPs. In order to achieve meaningful increases in protein-incorporated Met, the transgene needs to be highly expressed and the peptide/protein stable in the targeted tissue. Typically accumulation of foreign proteins is enhanced by targeting to the endoplasmic reticulum (ER) (Twyman et al., 2003). However, this can have the undesired consequence of overloading the protein folding and processing capacity of the ER (Oono et al., 2010; De Wilde et al., 2013).

Metabolic engineering of methionine biosynthesis is an alternative approach to increase methionine in the seed. The choice of which enzyme(s) to modify in which tissue(s) is complex and based on species-specific knowledge (or assumptions), such as where the methionine used in seed tissue for protein translation is synthesized, and in species capable of *de novo* Met synthesis in seeds, whether all steps of the sulfur assimilation pathway are also active in seeds as opposed to a pathway intermediate being transported into the seed. Additionally, one must consider if biosynthetic and transport pathways in other tissues can compensate for bottlenecks and limitations in the seed. The main assumption behind this metabolic engineering approach is that the pool size of free methionine and/or metabolic flux to methionine in the seed influence the profile of proteins synthesized.

Over the past several decades the general methods described above have been successfully used to substantially increase the methionine content of maize, soybean, and several other grain legumes (Molvig et al., 1997; Chiaiese et al., 2004; Song et al., 2013; Kim et al., 2014; Xiang et al., 2018; Amir et al., 2019). Although the quantity of rice used in livestock feed is currently dwarfed by other commodity crops (Global Rice Science Partnership, 2013), it is important to address improvement of rice protein quality. Among the major cereals, rice has the highest net protein utilization by livestock (Juliano, 1992). Furthermore, the regions of the world that cultivate maize and rice are globally distinct, and the development of rice varieties with increased methionine content would allow livestock farmers in rice-focused regions to reduce their reliance on blending higher methionine maize with soybeans for their livestock feed.

The sunflower seed albumin 2A, HaSSA, has attributes that make it an attractive choice for transgenic methods to study the effect of increased protein sink strength for sulfur amino acids (S-AA): it is remarkably dense in methionine and cysteine (16 and 8% by length, respectively) (Kortt et al., 1991), and it has been shown to be processed correctly in the seeds of several crops, which is important for transgene protein accumulation. Furthermore, it is rumen stable (Spencer et al., 1988; McNabb et al., 1994), making it suitable for blended sheep feeds, which need to be particularly rich in methionine for wool growth (Qi and Lupton, 1994). Based on positive results in lupin seed (Molvig et al., 1997), the *HaSSA* gene was introduced into rice under the control of a wheat glutelin (SSP gene) promoter and targeted to the endoplasmic reticulum (ER), where endogenous SSPs are post-translationally modified by disulfide bond formation and glycosylation (Hagan et al., 2003). By expressing HaSSA the goal was to “pull” additional S-AA, Met, and Cys into the seed protein fraction. Hagan and colleagues achieved high levels of HaSSA accumulation (to approximately 7% of total seed protein) in their transformed rice line, hereafter referred to as SSA (Hagan et al., 2003). At this level of accumulation, if the methionine incorporated into HaSSA was simply an additive to endogenous seed proteins at parental accumulation levels, then a 40% increase in total seed methionine would be expected in SSA seeds. However, only a 25% increase in total seed methionine was observed, and due to high intra-line variation this increase was not considered statistically significant (Hagan et al., 2003). In addition to the gap between predicted and observed change in total seed methionine, the profile of expressed proteins in SSA seeds was clearly different from the parental rice cultivar, Taipei 309 (Hagan et al., 2003). Several of the major changes were consistent with the hypothesis that free methionine became strongly limiting in the SSA seeds and the heterologous protein diverted free methionine away from endogenous protein translation. These results suggested that sink strength is not the primary factor limiting methionine content in rice seeds and that subsequent efforts in rice should focus on increasing the supply of methionine in the seed.

To address the assumed deficiency in methionine supply in rice, two enzymes in the methionine biosynthetic pathway have been targeted for modification: cystathionine-gamma-synthase (CGS) catalyzes the first of three enzymatic steps to synthesize methionine from cysteine (Hesse et al., 2004); serine acetyl-transferase (SAT) catalyzes the formation of O-acetyl serine (OAS) which provides the carbon backbone for cysteine, the thiol precursor for methionine (Watanabe et al., 2015). In rice it is not known to what extent methionine for protein synthesis is synthesized *de novo* in the seed and to what extent it (or a precursor) is imported from another tissue such as leaves. With this in mind, a ubiquitin promoter was chosen to drive expression of heterologous transgenes coding for these key enzymes in S-AA biosynthesis (Nguyen et al., 2012; Whitcomb et al., 2018).

In the case of CGS, a feedback-desensitized version of the enzyme from Arabidopsis (AtD-CGS) was chosen. Heterologous expression *AtD-CGS* in tobacco (Hacham et al., 2008; Matityahu et al., 2013), soybean (Song et al., 2013), and azuki bean (Hanafy et al., 2013) resulted in large increases in free methionine in vegetative tissues and in seeds. However, heterologous expression of *AtD-CGS* in rice did not result in increased methionine in leaves or seeds despite persistently elevated CGS activity in leaves (enzymatic activity in seeds was not tested) (Whitcomb et al., 2018). We suggested that flux to methionine was increased in the *AtD-CGS* transgenic lines (hereafter referred to simply as CGS), but in rice the concentration of free methionine may be homeostatically regulated and the additional synthesized methionine catabolized, similar to results for lysine (Karchi et al., 1994).

Ubiquitin promoter-driven expression of *cysE* from *E. coli* (*EcSAT*) was found to be more successful in rice than ubiquitin promoter-driven expression of *AtD-CGS* (Nguyen et al., 2012; Whitcomb et al., 2018). While the concentration of free methionine remained unchanged in the seeds of the *EcSAT* transgenic lines (hereafter referred to simply as SAT), total methionine was significantly increased in some of the generated lines. These results suggested that methionine accumulation in SSA seeds (Hagan et al., 2003) may not be limited primarily by insufficient methionine synthesis but rather by insufficient cysteine synthesis.

Here we generated double transgenic lines, containing both increased sink strength (“pull”) for methionine and increased cystine/methionine biosynthetic enzyme activity (“push”), to test whether combining these traits would result in a synergistic increase in rice seed methionine. This combinatorial approach has proved successful in maize, narbon bean, and potato (Demidov et al., 2003; Dancs et al., 2008; Planta et al., 2017), but to our knowledge, this is the first report to combine increased sink and source strength for methionine in rice. Specifically, we crossed SSA transgenic plants with the SAT transgenic line or the CGS transgenic line that we deemed most promising. Among the SAT rice lines generated by Nguyen et al. (2012), all had similarly high free cysteine (Cys) levels in their seeds. SAT47 was chosen for our “PushxPull” study because it showed the greatest increase in protein-incorporated methionine. Among the CGS lines generated by Whitcomb et al. (2018), CgSx4 was chosen because it had the highest measured CGS activity and marginally higher free methionine in seeds.

## Results

### Expression of *AtD-CGS*, *EcSAT*, and *HaSSA* Transcripts and Accumulation of HaSSA Protein in Seeds

**Figure 1:**
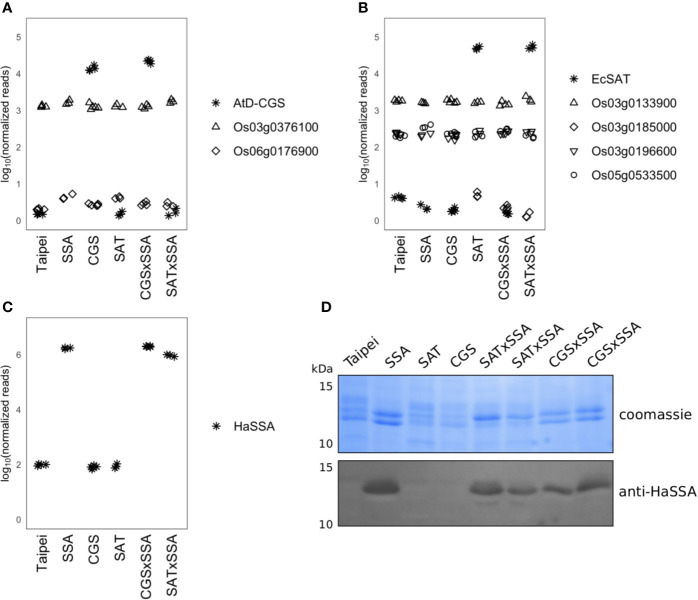
Expression of CGS, SAT, and SSA transcripts and accumulation of SSA protein in seeds. Normalized RNAseq read counts in milkripe seeds for **(A)** the *AtD-CGS* transgene and rice *CGS* isoforms, **(B)** the *EcSAT* transgene and rice *SAT* isoforms, and **(C)** the *HaSSA* transgene are plotted on a log_10_ y-axis to facilitate comparison of expression levels between transgenes and endogenous isoforms. Each symbol in the plotting area **(A–C)** represents the reads from the milkripe stage seeds of an individual plant. Biological replicates: Taipei n = 4, SSA n = 3, CGS n = 5, SAT n = 3, CGS × SSA n = 4, SAT × SSA n = 3. Significance testing is presented in **Supplemental Table 1**. **(D)** The SDS-soluble protein fraction from mature seeds was subjected to 15% acrylamide SDS-PAGE followed by western blotting with a polyclonal anti-SSA antibody. Each lane contains 20 μg of protein extracted from the seeds of an individual plant. The western blot was performed at least three times. The data shown are representative.

As a first step to characterize the seeds of the five transgenic lines used in this study we used RNA-seq data to determine transcript abundances of the *AtD-CGS* transgene and endogenous *CGS* genes, the *EcSAT* transgene and endogenous *SAT* genes, and the *HaSSA* transgene. The *AtD-CGS* and *EcSAT* transgenes are both driven by a maize ubiquitin promoter, and their transcripts were highly abundant in milkripe seeds, even after at least five generations post-transformation (**Figures 1A, B**). Considering only expressed genes in milkripe seeds, the *AtD-CGS* and *EcSAT* transgenes were expressed in the 97^th^ and 99^th^ percentiles, respectively, in this tissue. We identified one expressed endogenous *CGS* transcript (Os03g0376100, 76^th^ percentile) and three expressed endogenous *SAT* transcripts (Os03g0133900, Os03g0196600, and Os05g0533500, 81^st^, 21^st^, and 24^th^ percentiles, respectively) in milkripe seeds. The endogenous *CGS* transcript level was not significantly affected in any of the transgenic lines, but we did observe moderate upregulation of one of the endogenous *SAT* isoforms (Os05g0533500) in SSA seeds (1.6-fold relative to Taipei, padj < 0.001) and CGS × SSA seeds (1.4-fold relative to Taipei, padj < 0.02). We did not find any compensatory reduction in endogenous *CGS* or *SAT* transcripts in seeds expressing the *AtD-CGS* or *EcSAT* transgenes. These data show that at least one endogenous isoform of *CGS* and *SAT* is highly expressed in milkripe seeds. Therefore, transgene expression is likely to increase total enzymatic activity of the relevant methionine or cysteine biosynthetic step rather than to bring a new biosynthetic “trait” into the seeds.

Transcripts of the *HaSSA* transgene were also highly abundant in milkripe seeds (>99^th^ percentile) (**Figure 1C**), but as a sink for methionine and cysteine, HaSSA protein levels are of greater importance. Equal quantities of the SDS-soluble protein fraction from mature seeds were separated by SDS-PAGE and Coomassie stained (**Figure 1D**). Hagan et al., 2003 reported HaSSA accumulation equivalent to approximately 7% of salt extracted seed protein (Hagan et al., 2003), but we were not able to clearly identify the HaSSA protein band in SDS-solubilized seed protein by Coomassie staining alone. Identification of the HaSSA protein band was achieved by western blotting with a polyclonal HaSSA antibody. We observed a similar level of HaSSA protein accumulation in the CGS × SSA and SAT × SSA seeds, but it did not appear to be higher than in the parental SSA line (**Figure 1D**), despite the increased expression of the S-amino acid biosynthetic enzyme transgenes (**Figures 1A, B**).

### Seed Protein Profiles

The protein profile of SSA seeds is different from that of the parental Taipei as the abundance of several major SSPs is altered: reduction in glutelin acidic and basic subunits, reduction in alpha-globulin, and accumulation of prolamin 7/14 (Hagan et al., 2003; Islam et al., 2005). Since total S-AA and protein content of seeds was not significantly changed in SSA seeds, the authors concluded that production of HaSSA protein diverted the limited supply of free cysteine and methionine from endogenous relatively S-AA rich SSPs.

**Figure 2:**
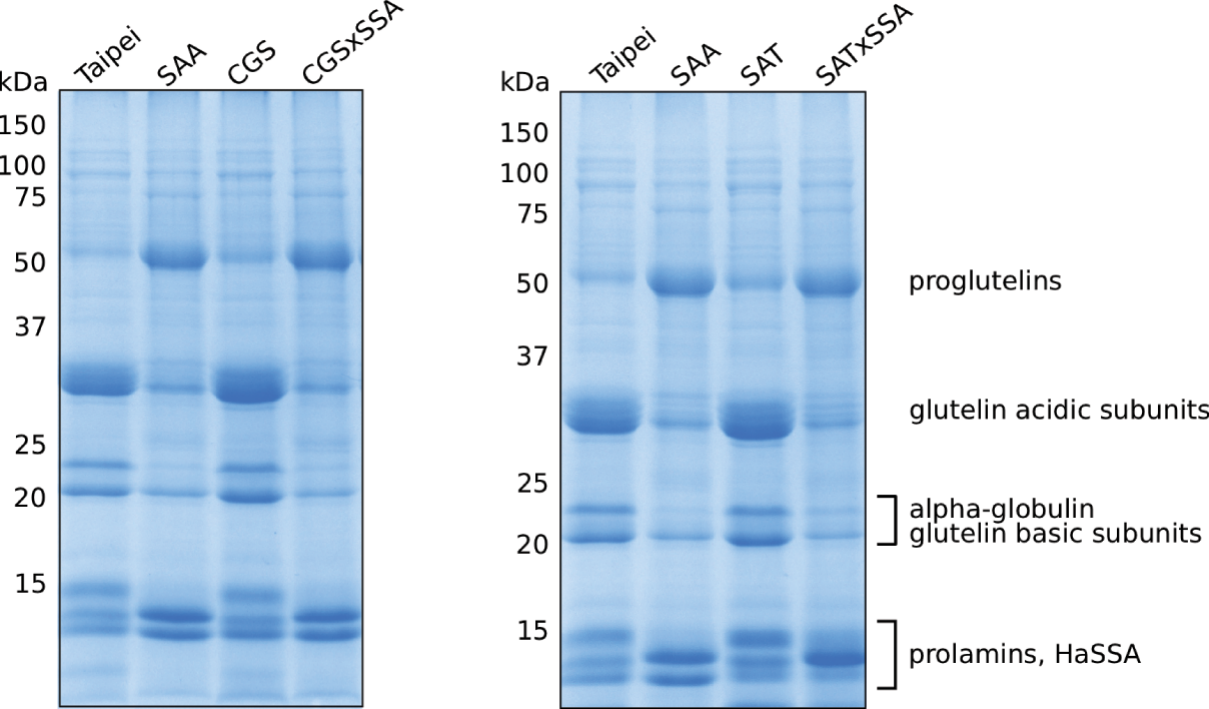
Seed protein profiles of mature seeds. The SDS-soluble protein fraction from mature seeds was subjected to 13% acrylamide SDS-PAGE followed by Coomassie staining. Each lane contains 10 μg of protein extracted from the seeds of an individual plant. Protein band labeling is based on previously published data (Hagan et al., 2003; Yasuda et al., 2009). The protein extraction and SDS-PAGE were performed three times. The data shown are representative.

Based on these data, we expected *EcSAT* and/or *AtD-CGS* to suppress the altered protein profile phenotype of *HaSSA* in double transgenic seeds. However, the protein profiles of CGS × SSA and SAT × SSA seeds look very similar to SSA (**Figure 2**). In contrast to SSA, the 1D SDS-PAGE protein profiles of SAT and CGS appeared indistinguishable from Taipei (**Figure 2**). These data suggest that either additional S-AA production in EcSAT- and AtD-CGS-containing lines is insignificant or other factors besides S-AA limitation contribute to the altered seed protein profile of HaSSA-containing seeds.

### Expression of Endoplasmic Reticulum Quality Control Genes

Other examples in rice seeds of highly expressed foreign proteins targeted to the secretory pathway have also resulted in significant changes in endogenous protein accumulation that partially overlap with those we observe in HaSSA-containing seeds (Takagi et al., 2005; Yasuda et al., 2005; Oono et al., 2010). Notably, the foreign proteins in these studies were not particularly rich in methionine or cysteine. More generally, strong expression of secretory pathway-targeted transgenes puts a heavy demand on the quality control machinery of the ER and can induce the unfolded protein response (UPR) in an attempt to maintain protein folding and modification fidelity as well as timely transport of proteins out of the ER to other destinations in the secretory pathway (Oono et al., 2010; De Wilde et al., 2013; Liu and Howell, 2016). The accumulation of glutelin precursors in the HaSSA-containing seeds (**Figure 2**) could indicate retention of proglutelin in the ER due to misfolding or insufficient trafficking of proglutelin out of the ER *via* COPII vesicles to the Golgi and eventually to protein storage vacuoles where it is proteolytically cleaved into acidic and basic subunits (Satoh-Cruz et al., 2010; Tian et al., 2013; Qian et al., 2015). Therefore, we investigated whether the altered protein profile in HaSSA-containing seeds may be associated with activation of an unfolded protein response (UPR) due to an overloaded ER protein processing machinery.

**Figure 3:**
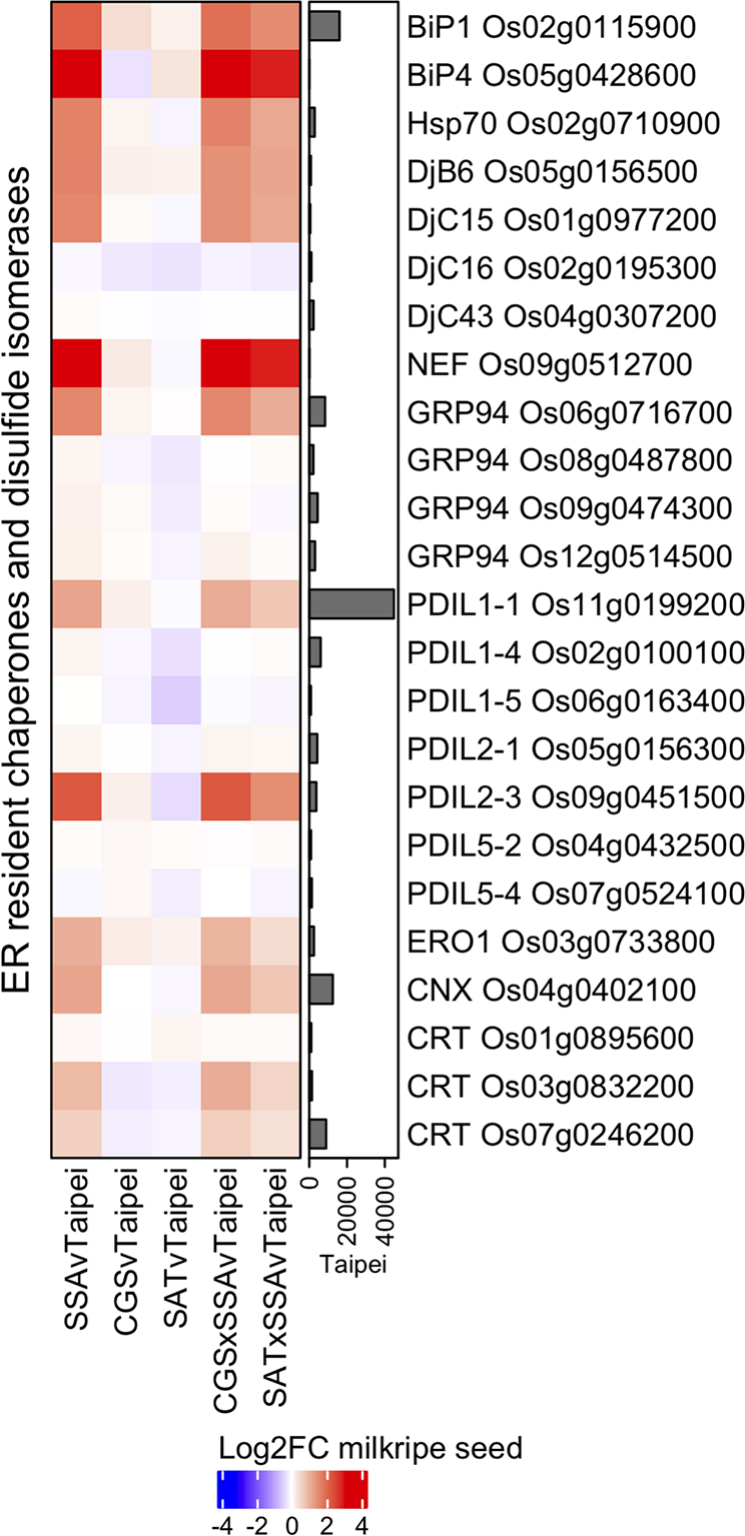
Differential expression of ER chaperones, co-chaperones, and protein disulfide isomerases in milkripe seeds. Differential expression data are presented in a heatmap matrix of Log_2_-fold change (Log2FC) relative to Taipei. The mean normalized read count in Taipei for each gene is presented in a bar graph annotation of the heatmap. Those ER resident chaperones (BiP, Hsp70, GRP94, CNX, CRT), co-chaperones (NEF, DjB, DjC), and protein disulfide isomerase (PDIL, ERO1) genes with a mean normalized read count > 1000 in at least one line are shown. Biological replicates: Taipei n = 4, SSA n = 3, CGS n = 5, SAT n = 3, CGS × SSA n = 4, SAT × SSA n = 3. Significance testing is presented in **Supplemental Table 1**.

Specific changes characteristic of the UPR include increases in ER resident chaperone, co-chaperone, and protein disulfide isomerase expression (Liu and Howell, 2016). The most abundant chaperone system in the ER lumen is composed of ATP-regulated Hsp70 family chaperones (BiP), ATP-independent co-chaperones of the Hsp40 family (DnaJ) and a nucleotide exchange factor. Additional important chaperones include the Hsp90 family (GRP94) and the calnexin/calreticulin (CNX/CRT) proteins. Protein disulfide isomerases (PDIs) mediate disulfide bond formation, dissolution, and reformation and are critical for protein folding. Differential expression analysis of these chaperones, co-chaperones, and protein disulfide isomerases revealed a general upregulation in seeds of the three HaSSA-containing lines and only minor expression changes in seeds of SAT and CGS lines (**Figure 3**). Further, the major isoform in milkripe seeds of each enzyme family (BiP1 Os02g0115900, GRP94 Os06g0716700, CNX Os04g0402100, CRT Os07g0246200, and PDIL1-1 Os11g00199200) is strongly and significantly upregulated specifically in the HaSSA-containing lines. Minor isoforms BiP4 Os05g0428600 and PDIL2-3 Os09g0451500, the nucleotide exchange factor for Hsp70 chaperones (NEF Os09g0512700), and the electron acceptor for PDI-mediated disulfide bond formation (ERO1 Os03g0733800) were also found to be upregulated, further supporting a functional increase in ER protein folding capacity in HaSSA-containing seeds. Notably, we observed little if any attenuation of UPR gene upregulation in the CGS × SSA and SAT × SSA seeds, as might be expected if S-AA limitation for HaSSA translation were the cause of the increased chaperone expression.

### Methionine, Cysteine, and Glutathione Levels in Mature Seeds

**Figure 4:**
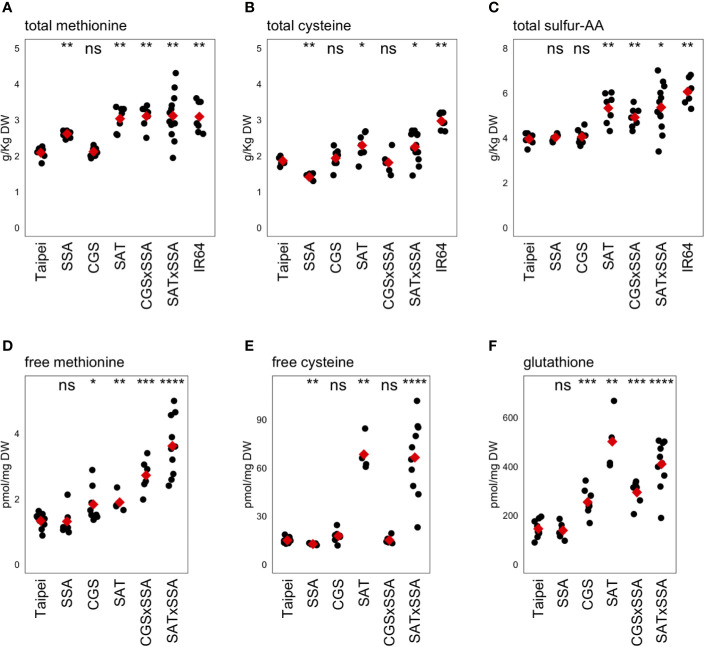
Methionine, cysteine, and glutathione levels in mature seeds. The concentrations of the sulfur-containing amino acids methionine **(A, D)** and cysteine **(B, E)** as well as the cysteine-containing tripeptide glutathione **(F)** were determined in mature seeds. The concentration of total sulfur-amino acids (S-AA) was determined by adding the total concentration of methionine and cysteine in the same seed sample **(C)**. Each black dot indicates the measured concentration in the seeds from an individual plant. The mean concentration in each line is indicated by a red diamond. The data presented are from two independent experiments. Wilcoxon rank-sum test was performed to compare the median concentration in each line to the median of Taipei. The significance level of each test is designated: ns (not significant) for p > 0.05, * for p ≤ 0.05, ** for p ≤ 0.01, *** for p ≤ 0.001, and **** for p ≤ 0.0001.

Published results from our group and others suggest that methionine content in rice is limited by both sink strength (the proportion of methionine codons in SSP transcripts) and by source strength (synthetic flux to S-AA) (Hagan et al., 2003; Lee et al., 2003; Nguyen et al., 2012; Whitcomb et al., 2018). Total seed methionine in SSA seeds was not significantly different from parental Taipei despite the strong accumulation of HaSSA protein (Hagan et al., 2003). We measured a comparable increase (25% compared to Hagan’s 30%), but due to much lower intra-line variation in our data, the calculated p-value (<0.005) was found to be highly significant (**Figure 4A**). Our selected SAT line also showed increased total methionine relative to parental Taipei, but this increase was lower than reported by Nguyen et al. (2012) (1.45 fold compared to 4.75 fold) (**Figure 4A**). This disparity may be due to a different technical method for determining total amino acid concentration in the seed. We chose here a standard method used in food quality control and employed a company to run the respective analyses. Consistent with Whitcomb et al. (2018), ubiquitin promoter-driven expression of feedback-desensitized *AtD-CGS* did not result in an appreciable increase in total seed methionine (**Figure 4A**).

The primary question of our study was whether push and pull traits would synergistically interact when combined in the same rice plant to allow increased accumulation of methionine in the seed. The data give a different answer to this question depending on which “push” trait is used: increased activity in the cysteine biosynthesis pathway, or farther downstream in the methionine pathway specifically. Combining *EcSAT* and *HaSSA* in seeds, both of which successfully increased total Met individually, did not lead to a further increase. However, co-expression of *AtD-CGS* with *HaSSA* in the seed was found to synergistically affect total seed methionine: CGS × SSA seeds had higher total methionine than either parent and higher than the sum of total methionine level in each parent, but accumulation was not higher than in SAT and SAT × SSA seeds. Three transgenic lines (SAT, SAT × SSA and CGS × SSA) had total methionine levels comparable to those in IR64, a “high protein” variety, reflecting an increase of approximately 50% over the parental Taipei line (**Figure 4A**).

In our transgenic lines, the magnitude of changes in total cysteine in the seed was found to be smaller than those for methionine (**Figure 4B**). The HaSSA protein is extremely methionine-rich (16% by length), but it also has a higher proportion of cysteine residues (8%) than most rice seed proteins. Despite this characteristic, total cysteine was actually lower in SSA seeds than in Taipei; the observed increases in total methionine seemed to come at the expense of total cysteine in this line. Hagan et al., 2003 also observed a decrease in total cysteine in SSA seeds. SAT catalyzes the formation of OAS, the carbon backbone for cysteine synthesis, and as expected, expression of *EcSAT* resulted in an increase in total cysteine in the seed (Hagan et al., 2003). Combining *HaSSA* with *EcSAT* relieved the decrease in total Cys found in SSA seeds but did not lead to a further increase above *EcSAT* alone (as is true for total Met), and although cysteine is a substrate for AtD-CGS, no decrease in total cysteine was observed in CGS seeds.

The observed increase in S-AA content in SAT × SSA and CGS × SSA seeds (**Figure 4C**) could be explained by especially high HaSSA levels, but based on the SSA western blot (**Figure 1D**), HaSSA protein accumulation in the double transgenic lines was if anything slightly lower than in SSA alone. The total S-AA data (**Figure 4C**) taken together with the seed protein profiles (**Figure 2**) and ER chaperone gene expression patterns (**Figure 3**) argue for an ER-stress based mechanism for seed protein profile change in HaSSA-containing seeds, rather than a redistribution of dramatically limited S-AA supply.

In rice seeds the proportion of S-amino acids that remain free rather than incorporated into polypeptides is extremely small. In the case of methionine, the average total pool size in Taipei was measured to be 2 g/kg dry weight (DW), which is equivalent to approximately 13,000 pmol/mg DW, while the average measured free methionine concentration was only approximately 1.4 pmol/mg DW. Highly significant differences in free methionine concentration were observed, but given how small the pool of free methionine is relative to the total pool size it is unclear whether these differences have any biological relevance (**Figure 4D**). While the total quantity of methionine and cysteine stored in the rice seed are very similar (both approximately 2 g/kg DW) (**Figures 4D, E**), the proportion that remains soluble is approximately 10-fold higher for Cys than for Met. We found a strong accumulation of free Cys in EcSAT-containing lines, as expected from literature. However, the level of accumulation was not attenuated by the presence of the strong sink for S-AA in the form of HaSSA protein (**Figure 4E**).

Glutathione (GSH), a tripeptide of cysteine, glutamic acid, and glycine, is a major metabolic product of cysteine, and is at least an order of magnitude more abundant in mature rice seeds than free cysteine. The two transgenic lines with the highest total cysteine and free cysteine, SAT and SAT × SSA, also had the highest GSH levels (**Figure 4F**). Interestingly, GSH also accumulated somewhat in AtD-CGS-containing lines, despite no increase in cysteine concentration, suggesting that increased cysteine synthesis occurs in AtD-CGS-containing seeds, but that it is diverted to downstream products such as GSH.

### Sulfate Levels and Sulfur Pools

**Figure 5:**
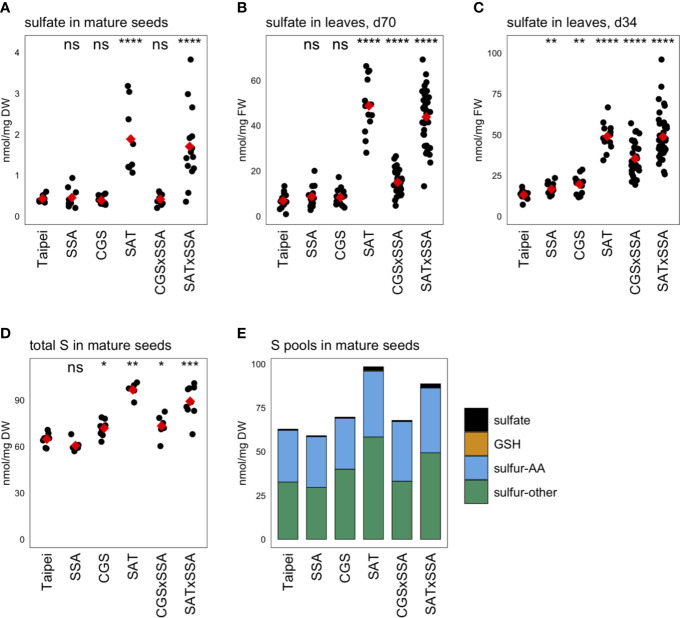
Sulfur pools. The concentration of sulfate was determined in **(A)** mature seeds and leaves from plants **(B)** 70 days (d70) after transplantation and **(C)** 34 days (d34) after transplantation. The total sulfur (S) in mature seeds was also determined **(D)**. Each black dot indicates the measured concentration in the specified tissue from an individual plant. The mean concentration in each line is indicated by a red diamond. The data presented are from two independent experiments. Wilcoxon rank-sum test was performed to compare the median concentration in each line to the median of Taipei. The significance level of each test is designated: ns (not significant) for p > 0.05, * for p ≤ 0.05, ** for p ≤ 0.01, *** for p ≤ 0.001, and **** for p ≤ 0.0001. For those mature seed samples for which total S, sulfate, GSH, total Met, and total Cys were determined, the mean concentration of each of these sulfur pools was calculated for each line. The S-other pool is the remainder of the sulfate, GSH, total Met, and total Cys concentrations subtracted from the total S concentration in each sample. The absolute and relative sizes of sulfur pools in mature seeds are visualized in a stacked bar graph **(E)**. Biological replicates for the S pools analyses: n = 12 for all lines except SAT × SSA, for which n = 28.

Unexpectedly, the sulfate concentration in mature seeds was found to be approximately four-fold higher in EcSAT-containing seeds than in all other lines (**Figure 5A**). SAT and SAT × SSA plants also had approximately four to seven-fold higher sulfate levels in their leaves (**Figures 5B, C**).

Our goal was to generate rice seeds enriched in S-AA. This either requires the seed to import more sulfur from other parts of the plant for assimilation into cysteine and methionine or to redistribute the existing sulfur pools to S-AA, or a combination of these two mechanisms. To assess whether redistribution of sulfur or additional import was driving the significantly elevated S-AA levels in SAT, SAT × SSA, and CGS × SSA seeds, we measured the total sulfur content. SAT and SAT × SSA seeds showed large increases in total sulfur (56 and 41% respectively). Although more subtle, CGS × SSA seeds also contained elevated total sulfur, and the size of that increase (13%) was comparable in size to the increase in S-AA in this line (**Figure 5D**). These data suggest that increased import of sulfur into the seed, rather than redistribution, underlies the increase in total Cys and Met in the seed of SAT, CGS × SSA, and SAT × SSA plants. In the case of EcSAT-containing seeds, the increase in total sulfur is substantially greater than the increase in S-AA, indicating that there are other sulfur-compound pools in these seeds that experience an increase. Although SAT and SAT × SSA seeds had large relative increases in sulfate and GSH, both major transport forms of sulfur, the magnitude of these pool size changes do not account for the large increase in total sulfur in these seeds. In fact, the *EcSAT* transgene had a larger effect on the size of the “sulfur-other” pool (sulfur not contained in the sulfate, GSH, or S-AA pools) than on sulfate, GSH, or S-AA (**Figure 5E**). Resolution of the identity of the differentially accumulating sulfur-containing metabolites in this pool was beyond the scope of our study and remains an interesting open question.

### Relative Transcript Abundance of Sulfate Transporters, S-Assimilatory Enzymes, and S-Methylmethionine Degradation Enzymes

**Figure 6:**
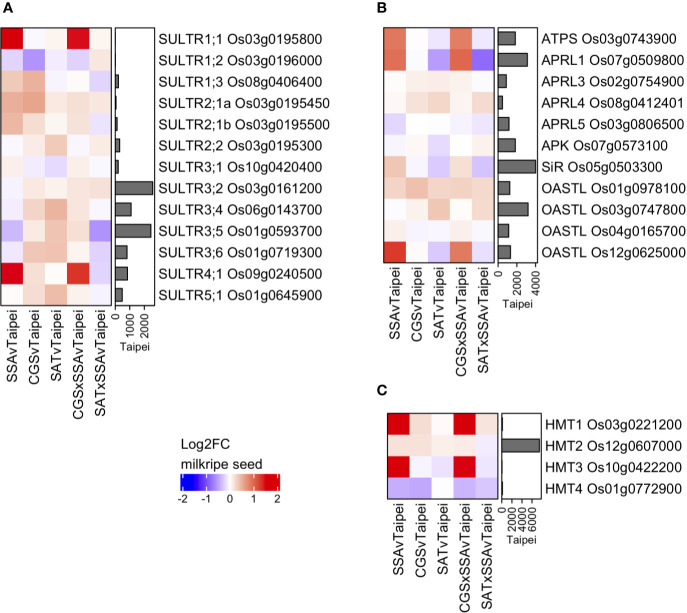
Differential expression of genes for sulfate transporters, S-assimilatory enzymes, and SMM degradation enzymes in milkripe seeds. Differential expression data are presented as heatmap matrices of Log_2_-fold change (Log2FC) relative to Taipei. The mean normalized read count in Taipei for each gene is presented in a bar graph annotation of the heatmap. All 13 genes annotated as sulfate transporters (SULTRs) in rice **(A)** and all four genes annotated as homocysteine methyltransferases (HMT) in rice **(C)** are shown. Annotated ATP sulfurylase, APR reductase, APR kinase, sulfite reductase, and OAS-thiol-lyase genes with a mean normalized read count > 500 in at least one line are shown **(B)**. Biological replicates: Taipei n = 4, SSA n = 3, CGS n = 5, SAT n = 3, CGS × SSA n = 4, SAT × SSA n = 3. Significance testing is presented in **Supplemental Table 1**.

Various S-compounds are known to be transported between tissues in different plant species (Gigolashvili and Kopriva, 2014). One of the most mobile S-compounds is sulfate. Sulfate transporters (SULTRs) are a family of proteins that can be classified into four groups based on specifics of their function and localization (Takahashi et al., 2011). The rice genome contains 13 SULTRs, the expression of which were determined in milkripe rice seeds in order to assess whether transcriptional regulation of these genes might provide evidence that increased sulfate import contributes to the observed differences in total sulfur in mature seeds (**Figure 6A**). Among the 13 rice SULTRs, we found the highest expressed were group 3 and group 4 transporters, which are responsible for intracellular sulfate transport (into plastids and out of vacuoles, respectively). High-affinity, plasma membrane-localized transporters in the SULTR group 1, which could be expected to contribute to sulfate import from source tissues, were notably expressed at much lower levels, with SULTR1;1 Os03g0195800 and SULTR1;2 Os03g0196000 close to the detection limit. This may explain the remarkably low level of sulfate in rice seeds. Three SULTRs were found to be differentially expressed in transgenic lines: SULTR4;1 Os09g0240500, SULTR1;3 Os08g0406400 and SULTR1;1 Os03g0195800. While the two group 1 SULTRs are likely plasma membrane-localized and were strongly and significantly induced, their absolute expression remained very low, and neither were upregulated in EcSAT-containing seeds. This suggests that additional S in these seeds is not due to increased transcription of plasma membrane localized transporters. However, these data do not exclude the possibility that SULTR activity may be differentially regulated post-transcriptionally or that sulfate flux into the seed may vary based on the concentration of sulfate in the phloem. In fact, the measured sulfate concentration in leaves from SAT and SAT × SSA plants was found to be 6 to 7-fold higher than Taipei (**Figures 5B, C**). Notably, we observed strong upregulation of SULTR4;1, but only in SSA and CGS × SSA (**Figure 6A**). SULTR4;1 has been shown to be localized to the tonoplast and is likely involved in sulfate efflux from this intracellular storage compartment (Kataoka et al., 2004; Zuber et al., 2010). Sulfate, whether imported across the plasma membrane or released from vacuolar storage, is reduced and assimilated into cysteine or adenosine 3′-phospho 5′-phosphosulfate (PAPS) in several enzymatic steps. Mirroring the differential expression pattern of SULTR4;1, at least one isoform of each enzyme in the sulfate assimilation pathway is transcriptionally upregulated in both SSA and CGS × SSA milkripe seeds: ATP sulfurylase (ATPS) Os03g0743900, adenosine 5′-phosphosulfate reductase 1 (APRL1) Os07g0509800, sulfite reductase (SiR) Os05g0503300, O-acetylserine (thiol) lyase (OASTL) Os12g0625000 (**Figure 6B**). Transcriptional upregulation of these enzymes and the vacuolar SULTR in SSA and CGS × SSA seeds could be the result of especially high unmet demand for reduced sulfate in these lines.

Another major inter-tissue transport form of sulfur is GSH (Kuzuhara et al., 2000). Two GSH transporters have been identified thus far in rice, OsOPT3 and OsOPT6 (Zhang et al., 2004; Wongkaew et al., 2018). OsOPT6 transcripts were below the detection limit in milkripe seeds, and OsOPT3 transcripts were expressed at an extremely low level (data not shown). Unless their expression strongly increases later in seed maturation, GSH is unlikely to be more than a very minor form of sulfur transported into the seed and cannot explain the differential accumulation of sulfur.

Two methionine-derived sulfur-containing metabolites, S-methylmethionine (SMM) and S-adenosylmethionine (SAM), are also potential forms of sulfur imported into the seed. SMM is readily detected in rice seedlings (Menegus et al., 2004), and the importance of SMM in inter-tissue sulfur transport has long been suggested (Bourgis et al., 1999; Tan et al., 2010; Cohen et al., 2017). Thus far there are no transmembrane transporters identified for SMM in plants (Gigolashvili and Kopriva, 2014), and therefore their expression pattern in seeds cannot be checked. But if increased SMM import underlies the differential accumulation of sulfur, then one might expect enzymes involved in SMM metabolism to be differentially regulated. Methionine can be generated from SMM and homocysteine in one step *via* the action of homocysteine S-methyltransferase (HMT). Of the four annotated HMTs in rice, two are strongly upregulated in SSA and CGS × SSA seeds, but are expressed at much lower levels than the major HMT isoform in seeds, HMT2, which showed only very minor expression changes in all our transgenic lines (**Figure 6C**). Whether upregulation of the minor isoforms, HMT1, and HMT3 results in higher flux of SMM to methionine in these seeds remains unclear, and the transcript data provide no evidence that differential SMM transport into the seed is relevant for the observed sulfur accumulation. SAM transmembrane transporters are represented by a family of seven genes in rice (GO:0000095). However, transcripts for all seven were expressed at only low levels in milkripe seeds, and none were deemed differentially regulated relative to Taipei (data not shown).

Taken together, expression patterns of S-compound transporters do not point to a specific S-compound that is increasingly imported into seeds with elevated S-AA contents. Of course, it remains possible that the import activity of specific S-compound transporters is increased in the absence of mRNA accumulation. Given the very high levels of sulfate in SAT and SAT × SSA leaves, these lines may have higher phloem concentrations of sulfate and thereby increased import of sulfate into the seed.

### OAS and OAS-Cluster Gene Expression in Milkripe Seeds

**Figure 7:**
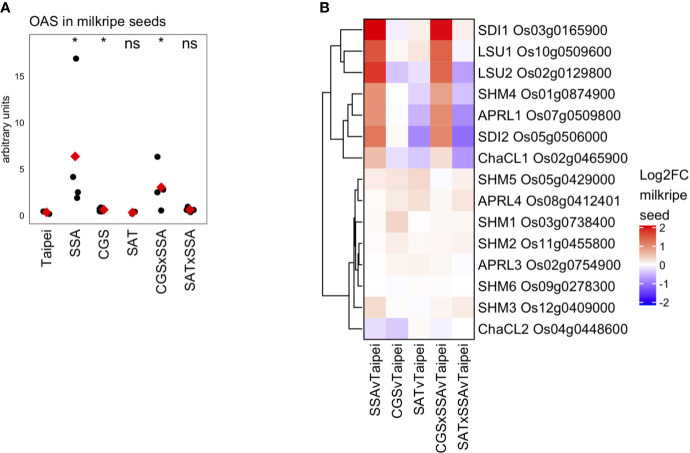
OAS accumulation and differential expression of OAS-cluster genes in milkripe seeds **(A)** The relative abundance (arbitrary units) of O-acetylserine (OAS) in milkripe seeds is presented. Each black dot indicates the determined relative abundance in the seeds of independent individual plants (n = 4). The mean abundance in each line is indicated by a red diamond. Wilcoxon rank-sum test was performed to compare the median accumulation in each line to the median of Taipei. The significance level of each test is designated: ns (not significant) for p > 0.05, * for p ≤ 0.05. **(B)** Differential expression data for the 15 putative orthologs in rice of the 7 so-called “OAS-cluster” genes in *Arabidopsis thaliana* are presented as a heatmap of Log_2_-fold change (Log2FC) relative to Taipei with genes clustered hierarchically using Euclidean distance metric and average linkage. Biological replicates: Taipei n = 4, SSA n = 3, CGS n = 5, SAT n = 3, CGS × SSA n = 4, SAT × SSA n = 3. Significance testing is presented in**Supplemental Table 1**.

OAS is often considered an indicator of sulfur deficiency or, alternatively, a signaling molecule responsive to sulfur deficiency. In the seeds of SSA and CGS × SSA lines exists an interesting situation where OAS (**Figure 7A**) and many transcriptional markers of sulfur deficiency (**Figure 6**) accumulate while the sulfate and total sulfur concentration in these seeds is not reduced relative to Taipei (**Figures 5A, D**). Since EcSAT catalyzes the formation of OAS, it was initially surprising that OAS does not accumulate in SAT seeds and that *EcSAT* expression suppresses HaSSA-associated OAS accumulation in the double transgenic seeds.

Hubberten et al., 2012 identified a cluster of six genes in Arabidopsis whose expression is highly correlated with OAS accumulation in a variety of conditions, including sulfur-deficiency and oxidative stress (Hubberten et al., 2012). The so-called OAS-cluster in Arabidopsis is composed of APR3, SDI1, SDI2, LSU1, SHM7/MSA1, and ChaC-like. With the exception of SDI1 and SDI2, multiple orthologs of each Arabidopsis gene are annotated in the rice genome. At least one ortholog of each OAS-cluster member was found in our data to be strongly and significantly upregulated in SSA and CGS × SSA seeds but not in SAT or SAT × SSA (**Figure 7B**), *i.e.* specifically in those lines with elevated OAS levels (**Figure 7A**).

## Discussion

### The HaSSA-Associated Seed Protein Profile May Result From Insufficient ER Folding Capacity Rather Than Insufficient S-AA Provision

We generated so-called “Push × Pull” lines by combining a strong S-AA sink with cysteine or methionine biosynthesis transgenes with the idea that increased production of S-AA would allow for high levels of HaSSA accumulation without downregulation of endogenous relatively S-rich SSP. However, the seed protein profile phenotype of SSA was not reverted to control composition by co-expression with either *AtD-CGS* or *EcSAT* (**Figure 2**), despite higher total S-AA levels in both double transgenic lines (**Figure 4C**). Specifically, total methionine was determined to be 20% higher in CGS × SSA and SAT × SSA seeds than in SSA (**Figure 4A**), and total cysteine was found to be 30 and 60% higher, in CGS × SSA and SAT × SSA, respectively, than in SSA (**Figure 4B**). These data suggest that the seed protein profile phenotype observed by Hagan et al. (2003) and Islam et al. (2005) in SSA rice seeds was not necessarily the result of insufficient S-AA supply and preferential incorporation into HaSSA.

Perhaps the most noticeable change in the SSA seed protein profile is to the glutelin component. Glutelins are referred to in Islam et al. (2005) as being relatively S-AA rich and their decrease in SSA seeds is used as evidence of S-AA redistribution. But with only 0.1–2.4% S-AA, we would instead characterize glutelins as having merely low or intermediate S-AA density, falling on the left side of the S-AA density distribution of SSPs and proteins coded for by the top 1% expressed transcripts in rice seeds (**Supplemental Figure 1**). Furthermore, it is not clear from data presented in Hagan et al. (2003) or Islam et al. (2005) whether the total glutelin content (proglutelin plus proteolytically cleaved glutelin subunits) of SSA seeds is altered. Given the lack of clear reduction in total glutelin levels and its low to intermediate S-AA density, we considered the possibility that accumulation of proglutelins and the reduction in glutelin subunits were symptoms of insufficient protein processing in the ER rather than a consequence of S-AA limitation. Direct reduction of the folding capacity of the rice endosperm ER has been achieved by deficiency in a primary chaperone in the ER lumen, PDIL1-1. In PDIL1-1-deficient seeds, proglutelin strongly accumulated and processed glutelin subunits were strongly reduced (Satoh-Cruz et al., 2010). Proglutelin also accumulated in seeds with a genetic block to secretory protein transport out of the ER to the Golgi *via* COPII vesicles (Tian et al., 2013; Qian et al., 2015).

In addition to differential accumulation of proglutelin and glutelin subunits, alpha-globulin levels are reduced in HaSSA-containing seeds. Unlike glutelins, alpha-globulin is S-AA dense, with 5.9% methionine and 4.3% cysteine by length (**Supplemental Figure 1**). But reduction in this S-AA rich SSP is also consistent with overloaded ER protein folding capacity due to high *HaSSA* expression. High expression of other foreign proteins and peptides targeted to the ER in rice seeds can produce seed protein profiles very similar to that of SSA, irrespective of the S-AA content of the highly expressed foreign protein. For example, transgenic rice seeds expressing dimers of 42 amino acid beta-amyloid peptides (Ab1-42) have strong accumulation in proglutelins and reductions in alpha-globulin levels despite the transgenic peptide containing zero cysteine residues and having only intermediate methionine density (2.4% by length) (Oono et al., 2010). Transgenic rice expressing seven tandem human T-cell epitopes to Japanese Cedar pollen (7Crp) also show increased proglutelins and decreased alpha-globulin (Takagi et al., 2005). The transgenic peptide contains zero methionines and is 3% cysteine by length. Importantly, the seed protein profile phenotype in 7Crp seeds depends upon the expression strength of the 7Crp peptide. At low expression levels, the seed protein profile looks very similar to untransformed seeds (Takaiwa et al., 2007), while at five-times higher expression, proglutelin accumulated and alpha-globulin was clearly reduced (Takagi et al., 2005). Not only were the seed protein profiles of the beta-amyloid and 7Crp rice seeds similar to SSA, CGS × SSA, and SAT × SSA, but a similar set of ER resident chaperones and co-chaperones is upregulated, including BiP1 Os02g0115900, BiP4 Os05g0428600, Hsp70 Os02g0710900, NEF Os09g0512700, GRP96 Os06g0716700, CNX Os04g0402100 and CRT Os03g0832200, presumably as a response to increased demand for folding capacity in the ER. Based on the data above, we propose that the seed protein profile phenotype in HaSSA-containing seeds is primarily a non-specific effect of ER protein folding capacity overload, rather than S-AA limitation.

Lupin seeds have also been targeted for high HaSSA accumulation (Molvig et al., 1997), and despite similar relative levels of exogenous protein accumulation (5% of total protein in lupin, 7% in rice), the authors did not observe a large change in the seed protein profile. This raises the question, is rice particularly susceptible to ER overloading? Rice has the lowest grain protein content amongst major cereals (Juliano, 1992) which perhaps limits the seed’s capacity to synthesize foreign proteins without producing stress in the ER. Rice seeds also have extremely low levels of available sulfur (in the form of sulfate) in comparison to some other crops (Molvig et al., 1997; Chiaiese et al., 2004; Shinmachi et al., 2010) which may prevent meaningful increases in GSH synthesis when there is greater than normal protein folding load in the ER. The precise role of GSH in the ER is still under investigation, but there are strong indications that GSH may be critical for reduction and PDI-mediated isomerization of non-native disulfide bonds (Chakravarthi et al., 2006). Misfolded proteins containing non-native disulfide bonds would be expected to accumulate in situations where the ER protein-folding machinery is under heavy load due to strong expression of ER targeted foreign transgenes such as *HaSSA*. Furthermore, some level of oxidative stress due to H_2_O_2_ production is intrinsic to disulfide bond formation in the ER (Harding et al., 2003). An overloaded ER that is not able to sufficiently correct and resolve misfolded proteins can result in H_2_O_2_ production significant enough to alter the glutathione redox state (Ozgur et al., 2014; Dietz et al., 2016) and to influence the cytosolic redox potential (Lai et al., 2018). Therefore, in the context of an overloaded ER protein folding machinery, it might be adaptive to specifically downregulate expression of SSPs with very high S-AA density, such as S-rich 10kDa prolamin (Hagan et al., 2003), so that cysteine could then be repurposed for GSH synthesis.

### HaSSA Provokes a Sulfur Deficiency-Like Molecular Phenotype in Rice Seeds That Is Suppressed by Co-Expression With *EcSAT* but Not *AtD-CGS*

In addition to the seed protein profile phenotype described above, we also found an association between the *HaSSA* transgene and a S-deficiency-like molecular phenotype (**Figure 6**, **Figure 7**), despite no reduction in total sulfur (**Figure 5D**). The strongest gene expression changes include upregulation of S-assimilation pathway genes SULTR1;1 Os03g0195800, SULTR4;1 Os09g0240500, ATPS 0s03g0743900, APRL1 Os07g0509800, SAT Os05g0533500, and OASTL Os12g0625000. OAS and the OAS-cluster genes (Hubberten et al., 2012) also accumulated specifically in the same set of lines. OAS has previously been shown to accumulate in chickpea seeds expressing high levels of HaSSA protein (Chiaiese et al., 2004). The HaSSA-associated S-deficiency molecular phenotype described here is presumably the result of signaling of especially high S-AA demand for seed protein translation or of ER protein folding overload and moderate oxidative stress, both of which generate a higher need for cysteine.

Unlike the seed protein profile phenotype, the main features of the HaSSA-associated S-deficiency molecular phenotype are suppressed by co-expression of *EcSAT* (**Figures 6** and **7**). Notably, OAS, the product of SAT enzymatic activity, does not accumulate in the seeds of EcSAT-containing plants (**Figure 7A**) despite very strong expression of the transgene and no compensatory reduction in endogenous SAT transcript levels in this tissue (**Figure 1B**). While initially surprising, these results are very similar to other published studies that also used a Cys-sensitive isoform of SAT as their transgene. Transgenic Arabidopsis that highly expressed a Cys-sensitive SAT from watermelon also had no OAS accumulation despite high measured SAT activity and increased GSH concentration, suggesting that additional OAS was synthetized but it was readily converted to Cys, which was then incorporated into GSH (Noji and Saito, 2002). Similarly, Hopkins et al. measured OAS levels in *EcSAT* (Cys-sensitive) transgenic potato that were similar to WT (Hopkins et al., 2005). Our EcSAT isoform is cysteine-inhibited and indeed the concentration of free cysteine is significantly higher in SAT and SAT × SSA seeds. This suggests that OAS production is essentially buffered by negative feedback at the level of EcSAT activity and additional OAS synthesized by EcSAT is quantitatively consumed by OASTL activity and converted to cysteine. Such complete conversion to cysteine of the additional OAS produced by the same *EcSAT* transgene has already been shown *in vitro* (Harms et al., 2000). OASTL Os030747800, the highest expressed OASTL isoform in milkripe seeds, is moderately yet significantly (padj < 0.0001) upregulated, specifically in SAT-containing seeds, perhaps facilitating the conversion of the additional OAS produced in these transgenic lines.

OAS is expected to accumulate in situations where the supply of sulfide is insufficient for the OAS available for formation of cysteine by OASTL. The production of sulfide from sulfate requires the contribution of eight electrons from reductants. As shown in **Figure 3**, seeds from all three of our HaSSA-containing lines showed signs of ER stress in the form of strong upregulation of UPR genes. Conditions that result in an overloaded protein folding machinery in the ER have been linked to perturbed redox status (Ozgur et al., 2014), and OAS is known to accumulate in situations of oxidative stress, such as menadione treatment (Lehmann et al., 2009). We propose that strong *HaSSA* expression may result in reduced availability of reductant (GSH and ferredoxin) for APR and SiR, a lower sulfide: OAS ratio than in non-HaSSA-containing seeds, and as a consequence, OAS accumulation. Furthermore, we suggest that the *EcSAT* transgene results in a large enough increase in sulfur import into the seed (**Figure 5D**) for the sulfate reduction pathway to produce enough sulfide to keep OAS from accumulating in SAT × SSA seeds (**Figure 7A**). Expression of the *AtD-CGS* transgene also increases S-import into the seed, but not enough to keep the flux to sulfide high enough for the additional S-AA demanded by HaSSA translation and folding. Flux from sulfate to sulfide may also be increased in SAT × SSA seeds compared to SSA and CGS × SSA due to additional synthesis of GSH (the electron donor for APR) (Bick et al., 1998) (**Figure 4F**) and ferredoxin (the electron donor for SiR) (Krueger and Siegel, 1982) made possible by a larger pool of synthesized cysteine.

Given the importance of thiol-based antioxidant systems in mitigating oxidative stress (Ulrich and Jakob, 2019), it is reasonable that sulfur assimilation, and cysteine synthesis in particular, would be enhanced under such conditions. In fact, the activities of both APR and SAT have been shown to increase during oxidative stress. The activity of the Arabidopsis APR1 isoform increases under oxidative conditions, probably *via* disulfide bond formation (Bick et al., 2001). Soybean plastidic SAT has been shown to be phosphorylated under oxidative stress, resulting in a loss of feedback inhibition by cysteine and increased activity (Liu et al., 2006). Although there is no evidence of SAT regulation by phosphorylation in Arabidopsis, the chloroplast cyclophilin CYP20-3 participates in folding of AtSAT1 in a redox-dependent fashion, to enhance the activity of AtSAT1 and to affect thiol contents (Dominguez-Solis et al., 2008). Additionally, it was recently shown that the availability of (reduced) GSH in Arabidopsis and tobacco positively affects S-assimilation downstream of APR activity, including the steady state concentration of cysteine and methionine (Cohen et al., 2020). The data presented in this study support that redox regulation of sulfate assimilation and cysteine synthesis also occurs in rice.

### Improving Nutritional Quality of Seeds

Using a set of S-AA “push”, “pull” and “Push × Pull” lines, we show that multiple paths can lead to higher seed methionine in rice. First, ubiquitin promoter-driven *EcSAT* is sufficient on its own to produce rice seeds with 50% higher total methionine content (**Figure 4A**). This demonstrated that there is an accessible path in rice seeds to higher methionine that does not require increasing the protein sink for methionine. This transgenic line was produced to “push” cysteine synthesis, but unexpectedly, we found that the transgene had the effect of increasing sulfur content in the seed (**Figure 5D**). As indicated above, rice seeds have very little available sulfate relative to some other cereals (Shinmachi et al., 2010) and grain legumes (Molvig et al., 1997; Chiaiese et al., 2004), and this characteristic may influence the capacity for increased accumulation of S-AA and GSH in the tissue. The relative contribution of increased SAT activity and increased sulfur supply to the resultant improvement in seed protein quality in SAT transgenic seeds remains to be investigated.

Second, while ubiquitin promoter-driven *AtD-CGS* and glutenin promoter-driven *HaSSA* are ineffective on their own, in combination they synergistically interact to result in seeds with higher seed methionine (**Figure 4A**). Like *EcSAT*, but to a lesser extent, the *AtD-CGS* transgene results in increased seed sulfur content, some of which is incorporated into cysteine (**Figure 5D**). AtD-CGS is ineffective on its own because the cysteine available as a substrate for CGS might be tightly regulated in the plastid and flux to GSH might be prioritized over methionine (**Figure 4F**). Translation of the large number of *HaSSA* transcripts produced in the developing seed has the potential to “pull” significant quantities of additional methionine into the seed. In fact, the *HaSSA* transgene has been successfully used to increase the methionine content of lupin seeds (Molvig et al., 1997) and chickpeas (Chiaiese et al., 2004). In both cases, total seed sulfur remained nearly constant, but the concentration of sulfate in the seed dropped significantly, suggesting that the additional methionine demanded by HaSSA protein accumulation required increased assimilation of the existing sulfate pool. In rice seed, the concentration of sulfate relative to total sulfur is remarkably low relative to both lupin and chickpea (approximately 1, 30, and 20% of total S in rice, lupin, and chickpea, respectively) (Molvig et al., 1997; Chiaiese et al., 2004). Introduction of the new sink HaSSA seems insufficient to induce S-AA biosynthesis in the seed or to trigger sulfate uptake. Further, the load HaSSA puts on the ER for its folding appears to generate significant stress, including oxidative stress. In particular, the high number of Cys residues in HaSSA may amplify the folding load since formation of incorrect disulfide bridges (which need to be resolved and the correct bridges formed) compounds with each additional cysteine in the peptide. Because oxidizing environments promote disulfide bond formation this effect may be especially strong when the ER is experiencing stress since the stress produces ROS. Dissolution of incorrect disulfide bonds oxidizes GSH to GSSG (Chakravarthi et al., 2006), reducing the pool of reductants available for sulfate to sulfide reduction for eventual Cys and Met amino acid synthesis. *AtD-CGS* and *HaSSA* are effective in combination because they relieve deficiencies in the other. *AtD-CGS*-mediated increase in S-import results in additional flux to Cys, which is further pulled into Met synthesis due to strong sink in the form in HaSSA (**Figure 4A**). In the context of this strong sink for Met, the relative prioritization of synthesized Cys for GSH synthesis is weaker. HaSSA-associated decrease in reductant supply for sulfate reduction is complemented by increased supply of S for assimilation to Cys and Met.

In contrast to *AtD-CGS*, when *EcSAT* is co-expressed with *HaSSA* it does not synergistically increase methionine content in the seed. In fact, the increase in total methionine and cysteine are nearly equivalent in both SAT and SAT × SSA. However, the seed protein profiles in these two lines look very different from each other, with the SAT × SSA seeds having the ER stress protein profile phenotype and SAT seeds having a profile very similar to untransformed Taipei (**Figure 2**). The additional S-AA in SAT seeds are likely distributed into many seed proteins, or into a few proteins with very high S-AA content that are expressed at low levels, both of which would make perceiving differences by 1D SDS-PAGE difficult. Taken together these results suggest that the total S-AA level is not a reliable determinant of the seed protein profile in rice.

### Limited Seed Sulfate Availability/Intake/Loading Limits S-AA Related Nutritional Quality in Rice

A common feature of all three transgenic lines in this study with increased S-AA content is elevated total sulfur in the seed. This indicates that sulfur loading is a factor that can limit grain cysteine and methionine content in rice and that the seed is able to assimilate additional sulfur into organic molecules including S-AA. However, we were unable to determine what form or forms of sulfur are differentially imported into high S-AA lines. Transcript data from milkripe seeds did not reveal clear candidates based on annotated transporter expression or expression of enzymes that would be responsible for metabolizing specific imported S-species into major branch point metabolites, such as cysteine. Some indirect evidence points to sulfate as being the major differentially imported form of sulfur. In EcSAT-containing lines sulfate levels were considerably higher than in Taipei in all tissues tested, i.e. leaves from plants 34 and 70 days after germination, and mature seeds (**Figures 5A–C**). And it was previously shown that total CGS activity (sum of activity from endogenous isoforms and *AtD-CGS* transgene) was positively correlated with sulfate content in leaves (Whitcomb et al., 2018). Therefore, phloem loading with sulfate may be increased in our S-AA “push” lines. Higher levels of sulfate in the phloem would likely translate into greater sulfate unloading into seed tissues, even in the absence of transcriptional upregulation of well-expressed plasma membrane-localized SULTRs in the seed. There is also evidence that SULTR activity can be regulated both post-transcriptionally and post-translationally (Hopkins et al., 2005; Rouached et al., 2005; Yoshimoto et al., 2007; Shibagaki and Grossman, 2010). OASTL has been shown to inhibit sulfate transport across the plasma membrane by direct interaction with the STAS domain of SULTR1;2 (Shibagaki and Grossman, 2010). OASTL also interacts with SAT, forming the cysteine synthase complex (Hell and Wirtz, 2011), and one could speculate that high EcSAT accumulation could bind-up enough OASTL to reduce OASTL binding with SULTR1;2 and thereby reduce OASTL-mediated inhibition of sulfate transport *via* SULTR1;2. In future studies, flux analysis with labeled S would be required to confidently identify the S-species that are differentially imported into EcSAT and to a lesser extent, AtD-CGS containing seeds.

In conclusion, the set of five transgenic lines presented here provides insight into the factors that limit cysteine and methionine accumulation in rice seed and suggest different approaches to produce even greater increases than those achieved here. First, seed sulfur loading is strongly implicated as a critical factor, although which transport form of sulfur is differentially imported is still unknown. Additionally, experiments to separate the effect of EcSAT’s serine acetyl transferase activity from its effect on seed sulfur loading should be performed in order to design the next iteration of rice transgenic lines. Notably, both *EcSAT* and *AtD-CGS* expression in our transgenic lines are driven by ubiquitin promoters. It would be helpful to know whether seed-specific or leaf – specific expression of these enzymes would be sufficient to increase import of S into the seed. Second, the very high expression level and S-AA density of HaSSA may actually inhibit its effectiveness as a method to “pull” S-AA in to rice seeds. The data here suggest that rice seeds possess insufficient protein folding capacity in the ER for the very high levels of HaSSA accumulation, which results in oxidative stress. Translation of *HaSSA* transcripts may capture enough synthesized cysteine (for translation and for conversion into methionine for translation) to shortchange other uses of cysteine, such as synthesis of GSH and other antioxidants that are particularly important in the context of accumulated unfolded proteins in the ER and altered redox state in other cellular compartments. Therefore, a SSP with more moderate S-AA density and/or expressed at more moderate levels may be more effective for increasing total methionine in rice seeds. For example, 2S albumin from sesame (S2SA) has approximately 3-fold lower S-AA density than HaSSA, but low copy number transformation resulted in a large increase in both total cysteine and methionine in rice seed and no major changes in the endogenous seed protein profile were observed (Lee et al., 2003). We interpret this as a sign that the seeds are experiencing less ER unfolded protein stress than the SSA rice line used here.

Improving the nutritive quality of commodity crops remains an important goal. In this study we provided a deeper understanding of the processes underlying rice seed protein accumulation and outlined a basis for overcoming hurdles to further increase protein-bound S-AA content to meet nutritional needs.

## Materials and Methods

### Rice Lines

The line Taipei, *Oryza sativa* ssp. *japonica* cv. Taipei 309, IRGC accession 42576, served as the untransformed control line for the single and double transgenic lines in this study. The line IR64, *Oryza sativa* ssp. *indica* cv. IR64, served as a high-protein reference line. Generation of the line SSA was previously described in Hagan et al. (2003). Briefly, Taipei was transformed by microparticle bombardment with a transgene construct containing the coding region of the 2S albumin SFA8 from sunflower, *HaSSA*, and the KDEL ER retention sequence driven by Bx17 wheat high molecular weight glutenin promoter. Generation of the line CGS was previously described in Whitcomb et al. (2018), and the line CgSx4 from that study is simply referred to here as line CGS. Briefly, Taipei was transformed by *Agrobacterium tumefaciens* with a transgene construct containing the coding region for a feedback desensitized variant of *Arabidopsis thaliana* cystathionine-gamma-synthase, *AtD-CGS* and the chloroplast-targeting transit peptide from *Pisum sativum*, driven by the maize ubiquitin 1 promoter. Generation of the line SAT was previously described in Nguyen et al. (2012), and the line SAT47 from that study is simply referred to here as line SAT. Briefly, Taipei was transformed by *Agrobacterium tumefaciens* with a transgene construct containing the *cysE* gene from *Escherichia coli*, *EcSAT*, and the Arabidopsis rbcS 5′ signal sequence for chloroplastic targeting, driven by the maize ubiquitin 1 promoter. The CGS × SSA and SAT × SSA lines were generated by crossing homozygous T2 SAT and CGS plants with the SSA line. In these crosses, SSA served as the pollen donor. F1 plants were screened by PCR for the presence of the *HaSSA* transgene. Double transgenic plants in the F2 generation were screened by PCR for both *AtD-CGS* and *HaSSA* or *EcSAT* and *HaSSA*, and two segregants from each cross were chosen for further analysis. Single seed decent was used to propagate the lines to the F3, F4, or F5 generation depending on the assay (see *Plant Material Sampling*). Data from the two lines derived from each of the initial Push × Pull crosses were analyzed separately and found to have substantively similar molecular and metabolic phenotypes. For visualization and significance testing, data from these lines were combined and presented as simply CGS × SSA or SAT × SSA. PCR primers for screening: AtD-CGS_F 5′ agg atc cgt ccg tca gct gag cat taa agc and AtD-CGS_R 5′ aaa gct tga tgg ctt cga gag ctt gaa g; EcSAT_F 5′ gac gct act caa gca cga aa and EcSAT_R 5′ ccc atc ccc ata ctc aaa tg; HaSSA_F 5′ atg gca agg ttt tcg atc gt and HaSSA_R 5′ att tgg cat ggt tgg gac at.

### Growth Conditions

Seeds were germinated at 28°C in the dark on tap water-soaked paper towels. After 10 days, seedlings were transferred to a growth chamber with the following conditions: 12 h day length with a photon flux density set at 600 μE m^−2^ s^−1^ (Lamps: Iwasaki Eye MT 400 DL/BH E40, DHL Licht, Wülfrath, Germany); 26°C in the light and 22°C in the dark; relative humidity of 75% in the light and 70% in the dark. Single seedlings were transplanted into individual pots (18 cm deep, 10 cm diameter) of waterlogged soil after seven days of acclimation to the growth chamber. Soil was a mixture of two parts potting soil (70% white moss peat, 30% clay), and one-part sand. One gram of slow-release fertilizer (Plantacote Depot 4 M; Lanxess, Langenfeld, Germany) and 0.1 g Fetrilon Combi (Compo, Münster, Germany) were mixed into the soil in each pot. Pots were kept partially submerged in water until seeds ripened, then all water was withdrawn, and the plants were allowed to dry out.

### Plant Material Sampling

Milkripe and mature seeds were harvested 10 days and 21 days after anthesis, respectively. Unfilled seeds and those with fungus-infected hulls were excluded. Milkripe seeds were flash frozen in liquid nitrogen, and mature seed samples were dried at 50°C for 2 days prior to storage at −80°C. Seed samples from double transgenic plants that were used for RNAseq and sulfur-containing metabolite analyses were of the F5 generation and the F4 generation, respectively. The 2^nd^ and 3^rd^ mature leaves on the main tiller were harvested at 34 and 70 days after transplantation and flash frozen. Leaf samples from double transgenic plants were from the F3 generation.

### Seed Protein Extraction and Western Blotting

100 mg finely ground mature seed samples were thawed on ice in 600 μl of protein extraction buffer [62.5 mM Tris-HCL pH 6.8, 2% SDS, 10% glycerol]. The protein concentration of the supernatant after centrifugation was determined by Bradford assay with BSA as a standard. Western blotting for HaSSA was performed with an anti-HaSSA polyclonal antibody kindly donated by Linda Tabe, CSIRO and an anti-sheep HRP-conjugated antibody from Thermo Scientific (Pierce).

### Total Sulfur Content

Whole mature seed samples with husks were milled with a Retsch Ultra-Centrifugal Mill ZM200 and dried overnight at 80°C. 250 mg aliquots of milled and dried sample were digested in 5 ml of trace analysis grade 65% nitric acid:perchloric acid (15:85, v/v) for 8.5 h at increasing temperatures [2 h at room temperature, 3 h at 60°C, 1 h at 100°C, 1 h at 120°C, 1.5 h at 175°C]. After cooling, 4 ml of 25% (v/v) nitric acid was added and the tubes were reheated to 80°C for 1 h. 13 ml of ultra-pure water was added, the solutions mixed well, and then heated again at 80°C for 30 min. After cooling, these solutions were brought up to 20 ml with 5% nitric acid (v/v) and filtered (Whatman, no. 42 from GE Healthcare). Solutions were analyzed by inductively coupled plasma optical emission spectroscopy (ICP-OES) on an Optima 7300 DV ICP-OES (Perkin Elmer LAS Ltd., Seer Green, UK) using appropriate quality control checks, *e.g.* calibration verification standards, in house standards, and certified reference materials.

### Total Methionine and Cysteine Content

Dry, mature seed samples of approximately 1 g were were oxidized with ice-cooled performic acid. Oxidation reactions were stopped with hydrobromic acid and then dried in a vacuum rotary evaporator. Residues were resuspended in 6N hydrochloric acid, and hydrolysis was allowed to proceed for 24 h at 110°C. After drying the samples in a vacuum rotary evaporator, the hydrolyzed residue was resuspended in demineralized water. The HPLC method for derivatization, separation, and quantification of methionine and cysteine content was performed essentially as previously described (Algermissen et al., 1989). Briefly, immediately prior to injection into the HPLC, samples were derivatized with ortho-phthaldehyde (OPA) in methanol with addition of borate buffer pH 9.5 and mercaptoethanol. HPLC was performed using a Merck LiChrospher^®^ 5 µm RP-18 100 Å, 250 × 3 mm ID column connected to an HPLC system from Agilent in combination with a fluorescence detector from Shimadzu. Acetate buffer solutions with different methanol concentrations were used as HPLC eluents for the separation of amino acids by a gradient method.

### Glutathione and Free Cysteine Concentration

50 mg aliquots of frozen, finely ground mature seed tissue were suspended in 500 μl 100 mM HCl, vortexed, and cleared by centrifugation. 60 μl of supernatant was then reduced by incubation at room temperature with 35 μl of 10 mM DTT in 100 μl of 0.25 M CHES, pH 9.4. The samples where then derivatized for 15 min in the dark with 5 µl of 25 mM monobromobimane in acetonitrile. The derivatization was stopped by adding 110 µl of 100 mM methanesulfonic acid, and the major soluble thiols were quantified by HPLC as previously described (Kreft et al., 2003). HPLC was performed using a Eurospher 100-5 C18, Column 250 × 4 mm connected to a Dionex HPLC system, and data were collected and processed with Chromeleon software version 6.8 from Dionex.

### Free Methionine Determination

50 mg aliquots of frozen, finely ground mature seed tissue were resuspended in 300 µl ice cold methanol followed by 15 min of shaking at 950 rpm at 70°C. As a second extraction step, 167 μl of chloroform was added, and samples were shaken at 950 rpm for 5 min at 37°C. 333 μl of water was vortexed into the samples, and then they were centrifuged for 5 min at 20,800 g. 100 μl aliquots of the upper polar phase were dried overnight in a vacuum concentrator. The dried polar phase was resuspended in 70 μl of 5 mM sodium phosphate buffer pH 6.2 and subjected to HPLC analysis with pre-column, online ortho-phthaldehyde (OPA) derivatization in combination with fluorescence detection (Lindroth and Mopper, 1979). HPLC was performed using a Phenomenex HyperClone™ 3 µm ODS (C18) 120 Å, LC column 150 × 4.6 mm connected to a Dionex HPLC system, and data were processed with Chromeleon software version 6.8 from Dionex.

### Sulfate Concentration Determination

Dried polar phase aliquots from methanol:chloroform extraction (see above, *Free Methionine Determination*) were resuspended in 1 ml ULC/MS grade de-ionized water, vortexed, and spun at 4°C for 15 min at full speed in a tabletop microcentrifuge. Sulfate concentration was determined by anion exchange chromatography using an ICS-3000 from Dionex. Data were collected and processed with Chromeleon software version 6.8 from Dionex.

### O-Acetyl Serine Determination

Metabolite profiling of milkripe seed tissue by gas chromatography–mass spectrometry (GC–MS) was performed essentially as previously described (Erban et al., 2007; Erban et al., 2019). To enrich for polar primary metabolites and small secondary products, 60 mg samples were extracted with methanol and chloroform as described above for free methionine determination but with the following changes for GC–MS analysis: extraction volumes were scaled to the larger initial sample mass, ^13^C_6_-sorbitol at 17 mg/L was added to the methanol extraction step to allow later correction of analytical variance, and 160 μl, rather than 100 μl, polar phase aliquots were taken for analysis. Dried polar phase aliquots were chemically derivatized by methoxyamination and trimethylsilylation. A mixture of n-alkanes was added to the derivatized samples to serve as retention index standards. Gas chromatography coupled to electron impact ionization time-of-flight mass spectrometry was performed using an Agilent 6890N24 gas chromatograph and a Pegasus III mass spectrometer from LECO Instruments. ChromaTOF software (LECO, St. Joseph, MI, USA) was used to process the chromatograms. For metabolite identification TagFinder (Luedemann et al., 2008), NIST mass spectral library and search software (https://www.nist.gov/srd/nist-standard-reference-database-1a-v17), and the mass spectral and retention time index reference collection of the Golm Metabolome Database (http://gmd.mpimp-golm.mpg.de/) were used. Data were annotated and curated manually. Mass spectral intensity was normalized to sample fresh weight and ^13^C_6_-sorbitol. The twice silylated (2TMS) derivative of O-acetylserine (MPIMP ID, A141001; http://gmd.mpimp-golm.mpg.de/Analytes/b4a42a07-e58c-4fb3-83e5-21fc5dc3330a.aspx) was chosen for relative quantification of OAS levels in milkripe seeds.

### Differential Gene Expression Analysis

Milkripe seed samples were harvested 10 days after flowering on an individual panicle basis. Total RNA was prepared from approximately 100 mg of frozen, finely ground tissue with Ribospin™ Seed/Fruit kit (GeneAll Biotechnology, Korea) followed by two rounds of DNase treatment (first round with Riboclear™ plus! from GeneAll, second round with TURBO DNA-*free* Kit from Invitrogen), and PCR was performed to assess possible remaining genomic DNA contamination. Only those total RNA samples with RIN quality scores greater than 7.3 by Bioanalyzer were submitted for RNA sequencing. Library preparation and sequencing were performed at the Max Planck Genome Center, Cologne, Germany (https://mpgc.mpipz.mpg.de/home). rRNA depletion was performed on 1,000 ng total RNA samples with the Ribo-Zero™ rRNA Removal Kit (seed/root) from Illumina. Subsequent library preparation was performed with NEBNext^®^ Ultra™ Directional RNA Library Prep Kit for Illumina^®^ (New England Biolabs) according to the manufacturer’s instructions. Quality and quantity were assessed at all steps *via* capillary electrophoresis (TapeStation, Agilent Technologies) and fluorometry (Qubit, Thermo Fisher Scientific). Libraries were immobilized and processed onto a flow cell with cBot (Illumina) and subsequently sequenced on HiSeq3000 system (Illumina) with approximately 50 × 10^6^ strand-specific 150 bp single-end reads for each milkripe seed sample. The quality of the raw fastq sequence data was assessed by FastQC (http://www.bioinformatics.babraham.ac.uk/projects/fastqc/). Raw data were mapped first against the rice genome and then against a set of transgenes. In detail, sequence data were mapped using STAR (Dobin et al., 2013; Dobin and Gingeras, 2015) version 2.7.1a with the parameters –quantMode TranscriptomeSAM GeneCounts, –outSAMtype BAM Unsorted, and –outReadsUnmapped Fastx. Ensembl version 43 (IRGSP 1.0) genome reference in FASTA format (ftp://ftp.ensemblgenomes.org/pub/release-43/plants/fasta/oryza_sativa/dna/Oryza_sativa.IRGSP-1.0.dna.toplevel.fa.gz) and Ensembl version 43 cDNA Annotation in GTF format (ftp://ftp.ensemblgenomes.org/pub/release-43/plants/gtf/oryza_sativa/Oryza_sativa.IRGSP-1.0.43.gtf.gz) were used for genome indexing (–sjdbOverhang 149 –genomeSAindexNbases 13). In order to determine RNA read counts for the *AtD-CGS*, *EcSAT*, and *HaSSA* transgenes, reads unmapped to the rice genome were used as input for mapping against a fasta file containing the sequences X56686.1, M15745.1, and NM_110977.3, downloaded from NCBI), which was indexed with bwa version 7.17 in “is” mode. Reads were mapped against it in backtrack (“aln”) mode (Li and Durbin, 2009) and converted to sam format with bwa samse. Afterwards samtools version 1.8 was used to convert sam to bam format (samtools view -bS), sort (samtools sort), and index (samtools index) the resulting bam alignment files in order to create read counts per input sequence (samtools idxstats) (Li et al., 2009). Anti-strand read counts from the ReadsPerGene files of all 22 samples were merged in order to perform differential expression analysis. Reads that could be unambiguously mapped to either a rice gene or transgene were retained for further analysis in R version 3.6.1 with the package DESeq2 (Love et al., 2014) version 1.24.0. Differences in sequencing depth between samples were accounted for in DESeq2 with median-of-ratio normalization. Differential expression analysis was performed using dispersion and log fold change estimates after shrinkage with the DESeq2 *maximum a posteriori* option. To determine the significance level of estimated Log_2_-fold change, Wald tests were performed, and genes were considered differentially expressed relative to Taipei if the Benjamini–Hochberg adjusted p-value was less than 0.01. Genes were considered “expressed” in milkripe seeds if their average normalized counts were greater than 100. The empirical cumulative distribution function ecdf from the R stats package version 3.6.1 was used to calculate the expression strength percentile among all “expressed” genes in milkripe seeds.

### Data Visualization

Quantitative data visualizations were prepared in R version 3.6.1. Univariate scatter plots and the stacked bar plot were generated with the R package ggplot2 version 3.2.1. Significance testing results were added to the plots with the R package ggpubr version 0.2.3. Heatmaps were prepared with the R packages ComplexHeatmap version 2.0.0 and circlize version 0.4.7.

## Data Availability Statement

The RNA-seq dataset generated for this study has been deposited in NCBI's Gene Expression881 Omnibus (Edgar et al., 2002) and are accessible through GEO series accession number GSE149252882 (https://www.ncbi.nlm.nih.gov/geo/query/acc.cgi?acc=GSE149252).

## Author Contributions

RH and SW contributed to the conception and design of the study. SW, AR, and FB grew all plants, harvested samples, and processed them for analytical measurements. FB performed the crosses and selected transgenic lines. SP and MH performed the ICP-OES analysis. AE and JK performed analysis of the GC–MS data. SW and AF performed the RNA-seq analysis. SW performed the statistical analysis and prepared all visualizations. SW took the lead in interpreting the data and wrote the first draft of the manuscript. All authors contributed to the article and approved the submitted version.

## Funding

The primary funding sources for this study were the Max Planck Society (Germany) and the EU RTN BIONUT (BIOchemical and genetic dissection of control of plant mineral NUTrition) FP7: 264296. Rothamsted Research receives grant-aided support from the Biotechnology and Biological Sciences Research Council (BBSRC) through the Designing Future Wheat programme [BB/P016855/1].

## Conflict of Interest

The authors declare that the research was conducted in the absence of any commercial or financial relationships that could be construed as a potential conflict of interest.

## Supplementary Material

Supplementary Table 1 Significance testing results for genes presented in **Figures 1**, **3**, **6**, and **7**.
