# Supplementary material for: Cysteine and Methionine Biosynthetic Enzymes Have Distinct Effects on Seed Nutritional Quality and on Molecular Phenotypes Associated With Accumulation of a Methionine-Rich Seed Storage Protein in Rice
Source: Front Plant Sci. 2020 Jul 22;11:1118. doi: 10.3389/fpls.2020.01118 (PMC7387578; doi:10.3389/fpls.2020.01118)
Supplement: Supplementary file 1 [file Image_1.pdf]

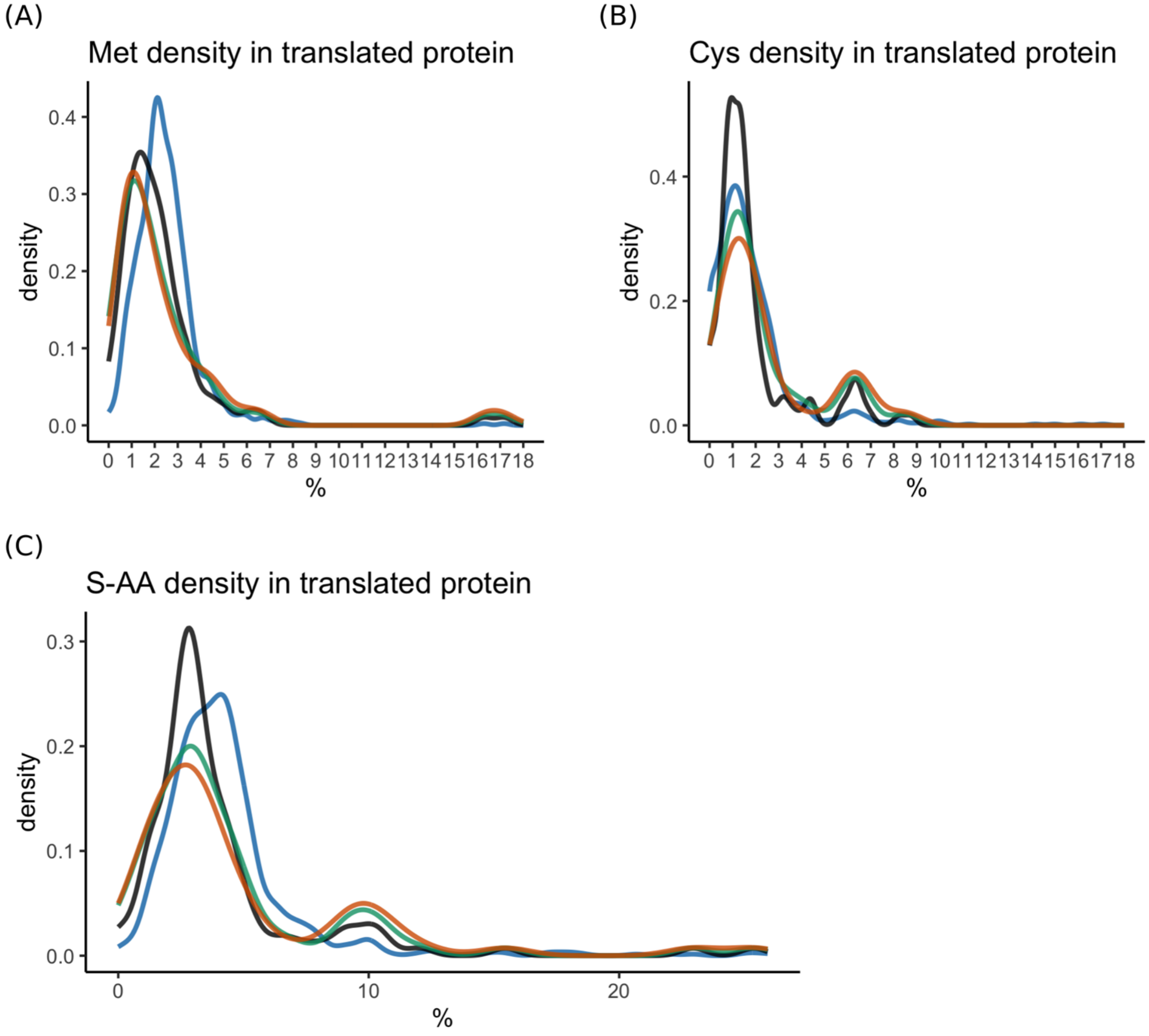

Supplementary Figure 1. Distribution of methionine, cysteine, and sulfur amino acid density in seed proteins. UniprotKB identifiers and protein sequences for selected genes were retrieved in batch using Uniprot.ws R package version 2.24.1 with taxId = 39947 to specify *Oryza sativa japonica*. The protein length and the total number of methionine and cysteine residues in the proteins were tabulated in order to determine the density of the respective amino acid in each protein (% by length). The distribution of % methionine **(A)**, % cysteine **(B)**, and % sulfur amino acids **(C)** were plotted for proteins in the following sets: seed storage proteins, 114 in set (black); seed storage proteins coded for by RNAs with mean expression greater than 100 reads, 70 in set (green); seed storage proteins coded for by RNAs with mean expression greater than 1000 reads, 53 in set (orange); and proteins coded for by the RNAs with expression in the top1% of all those measured by RNA-seq, 510 in set (blue).
